# Stand-alone extreme lateral interbody fusion (stand-alone XLIF) to treat radicular symptoms in patients with lumbar degenerative scoliosis: A monocentric observational study

**DOI:** 10.1016/j.bas.2025.104321

**Published:** 2025-07-05

**Authors:** Carolin Albrecht, Maximilian Schwendner, Paul Backhaus, Vicki M. Butenschoen, Bernhard Meyer

**Affiliations:** a Department of Neurosurgery, Technical University of Munich, TUM School of Medicine and Health, Klinikum Rechts der Isar, Germany; b Department of Neurosurgery, Heidelberg University Hospital, Germany

**Keywords:** Stand-alone XLIF, XLIF, Scoliosis, Extreme lumbar interbody fusion, Adult degenerative scoliosis, Spondylolisthesis

## Abstract

**Introduction:**

Extreme lateral interbody fusion (XLIF) is commonly used for scoliosis and spondylolisthesis in conjunction with posterior spinal fixation. Stand-alone XLIF may serve as an intermediate strategy for radicular symptoms in neuroforaminal or spinal canal stenosis with severe coronal imbalance, avoiding extensive posterior fixation in frail patients. This study evaluated its efficacy in treating radicular symptoms in degenerative scoliotic patients without posterior instrumentation.

**Material and methods:**

We retrospectively analyzed 19 patients who underwent stand-alone XLIF and dorsal decompression if required between January 2021–June 2024 for degenerative stenosis due to thoracolumbar scoliosis or listhesis with coronal deformity. Outcomes included symptom relief, revision surgery and radiological features like foraminal height restoration. We correlated initial diagnosis and fused levels with success rates to identify predictive factors.

**Results:**

Patients ranged from 65 to 86 years, 47 % were male and 53 % female. Most (42.1 %) underwent single-level fusion; 31.6 % had up to three levels fused. Radicular symptom relief was achieved in 52.6 % of patients following stand-alone XLIF. An additional 36.8 % experienced symptom relief after secondary dorsal decompression resulting in an overall relief rate of 89.5 %. Two patients (10.5 %) required secondary posterior instrumentation. Complications included retroperitoneal hematoma and cage dislocation.

**Discussion and conclusions:**

After stand-alone XLIF, 89.5 % of patients achieved pain relief. This procedure addresses neuroforaminal stenosis via indirect decompression and supports secondary fusion, reducing the need for extensive corrective spondylodesis. It is a viable option for frail patients with degenerative scoliosis and radicular symptoms. However, no long-term follow-up was performed and conclusions regarding durability are limited.

## Highlights

• Stand-alone XLIF achieved 90 % symptom relief in frail scoliosis patients.
• Reduced operative time, blood loss, and hospital stay with Stand-alone XLIF.
• In most patients posterior instrumentation could be avoided.
• Secondary direct decompression may further lead to reduced radicular symptoms.

## 1 Introduction

Adult degenerative scoliosis (ADS) is characterized by a spinal curvature greater than 10° in individuals who have reached skeletal maturity (Ploumis et al., 2007). The gradual degeneration of intervertebral discs in the aging individual can lead to uneven spine collapse, resulting in a thoracolumbar coronal plane deformity. This process is often exacerbated by disc bulging, facet joint arthritis, and thickening of the ligamentum flavum, which contribute to asymmetric disc collapse and subsequent neural compression (Ploumis et al., 2007). Neurogenic claudication and radicular symptoms are common clinical manifestations associated with ADS. Recent studies indicate an increase in the prevalence of these conditions, a trend associated with the aging population (Watanuki et al., 2012).

Posterior spinal fusion with pedicle screws combined with sagittal and coronal balance correction with osteotomies is a widely used approach to treat ADS, as well as spondylolisthesis (An et al., 2023). Extreme lateral interbody fusion (XLIF) or anterior lumbar interbody fusion (ALIF) often provides anterior support (Malham et al., 2012). XLIF is specifically designed to reduce the need for osteotomies in elderly patients who often suffer from comorbidities such as osteoporosis or require anticoagulant therapy (Rodgers et al., 2010, 2011). These patients are at increased risk for complications, including intra- or post-operative bleeding and protracted recovery. In addition, the increasing adoption of XLIF is largely due to its shorter operative time and fusion rates of 85.0 %–93.3 % in one year, underscoring its high efficacy (Malham et al., 2012; Manzur et al., 2020).

Stand-alone XLIF (SA-XLIF) may present an intermediate surgical strategy for primarily radicular symptoms for neuroforaminal or spinal canal stenosis in cases of degenerative coronal imbalance to avoid extensive posterior dorsal fixation and correction procedures, as those techniques are often associated with a longer operation time, higher blood loss and longer hospital stay (Meredith et al., 2013; Berjano et al., 2015) as well as higher revision rates. The technique was first introduced in 2001 by Pimenta (Pimenta, 2001; Ozgur et al., 2006) to place a large cage within the disc space to form a stable construct that distributes load forces evenly across the entire endplate. In ADS, this prevents the spine from further rotating. In addition, cage insertion helps to achieve indirect decompression of the foraminal space by restoring the foraminal height, which is determined by the cage size and angle. Furthermore, using cages with different angulations allows for correcting coronal deformities during the procedure.

Previous research suggests that SA-XLIF may be viable for treating neurogenic claudication and radicular symptoms (Hansen et al., 2015; Chen et al., 2019). Hansen et al. demonstrated that SA-XLIF significantly reduced leg and back pain in a cohort of 22 consecutive patients, with improvements observed at one- and two-year follow-up (Hansen et al., 2015). However, with a revision rate of 31.8 %, the authors recommend the addition of posterior instrumentation, which compromises the inherent benefits of the SA-XLIF technique (Hansen et al., 2015).

This study aims to evaluate the efficacy of SA-XLIF in patients with neurogenic claudication and/or radicular symptoms associated with adult spondylolisthesis or ADS. Specifically, we want to determine if invasive posterior instrumentation can be avoided in these predominantly elderly patients to reduce operative time, blood loss, and length of hospital stay. Furthermore, we hypothesize that performing SA-XLIF without the need for extensive posterior instrumentation and muscle preparation to place screws may result in a lower incidence of adjacent segment degeneration (ASD). In addition, it can lead to a rapid recovery which allows the patient to quickly return to his or her normal daily activities. Furthermore, we consider and analyze SA-XLIF as a potential first step in a two-stage approach in which secondary dorsal decompression can be indicated in patients with mild persistent symptoms after initial SA-XLIF.

## 2 Material and methods

### 2.1 Study design and patient cohort

We performed a retrospective monocentric study analyzing all patients treated for degenerative neuroforaminal stenosis and/or central canal stenosis due to thoracolumbar ADS and/or spondylolisthesis with coronal deformity between January 2021 and May 2024.

The concept involved a two-stage procedure: (1) stand-alone SA-XLIF and (2) dorsal decompression without instrumentation, if required.

Patients were excluded if they had undergone posterior fusion at the corresponding levels before XLIF or if they underwent preplanned posterior fusion during the same hospital stay. The study was conducted in line with the principles of the Declaration of Helsinki. Due to its retrospective design, informed consent was not necessary.

### 2.2 Outcome parameters

Postoperative symptom relief after SA-XLIF or SA-XLIF with subsequent posterior decompression was the primary outcome parameter.

The clinical improvement was assessed retrospectively based on documented symptom relief (yes/no) during standardized physical examinations performed immediately after surgery and before planned discharge. The need for secondary posterior fixation due to persistent symptoms was used as an objective indicator of insufficient clinical improvement.

Secondary outcome measures included the need for reoperation with posterior instrumentation, complications, operative time, intraoperative blood loss, and length of hospital stay. Additionally, radiographic characteristics were evaluated, particularly the restoration of foraminal height measured by the change in Cobb angle. We examined the relationships between initial diagnosis, number of fused vertebral levels, and success rates to identify predictive factors associated with favorable outcomes.

Regarding follow-up, all patient records were reviewed retrospectively in November 2024 to assess for re-admissions and re-operations providing a minimum follow-up period of six months following the last included patient in May 2024. However, no routine outpatient follow-up was scheduled as part of the study protocol.

### 2.3 Statistical analysis

Statistical evaluation was performed using SPSS (IBM SPSS Statistics, version 29.0.1.0). Data are reported as mean ± standard deviation (SD) for normally distributed variables. For non-normally distributed data, descriptive statistics such as median and interquartile range (IQR) or minimum and maximum values are provided. Statistically significant differences were assessed using the student's t-test, chi-square test, or paired *t*-test for parametric data and the Mann-Whitney test for non-parametric data. Statistical associations were analyzed using the logistic regression models. The accepted level of significance for all tests was P < 0.05.

## 3 Results

### 3.1 Patient characteristics

**Fig. 1:**
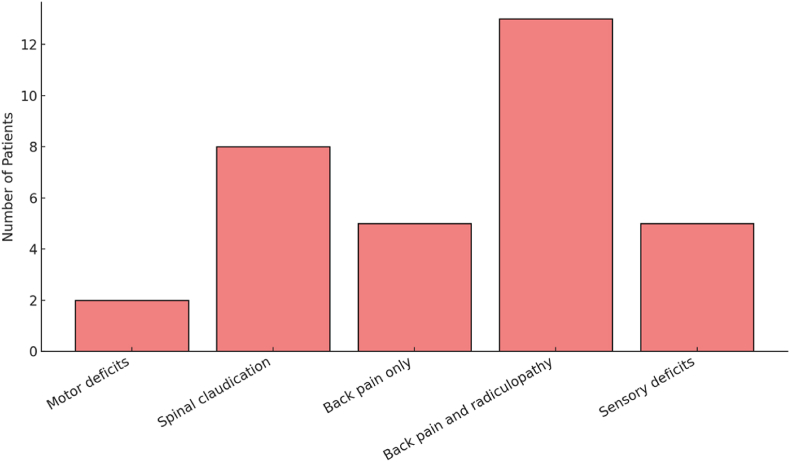
Symptom distribution among patients. Note that some patients experienced multiple symptoms. All patients suffering from motor and sensory deficits experienced additional radiculopathy in the affected dermatome(s) with no substantial back pain reported.

Between January 2021 and May 2024, 19 patients were operated for radicular symptoms attributed to ADS using stand-alone XLIF. Of these patients, 53 % were female and 47 % male with a median age of 78 years (ranging from 65 to 86 years). Twelve patients (63 %) had a history of prior lumbar spine surgery, primarily minimally invasive decompressive procedures but also posterior instrumentation at adjacent levels in 10.5 % of the cases. The indications for SA-XLIF were as follows: All 19 patients were diagnosed with adult degenerative scoliosis (ADS). Of these, seven presented with central canal stenosis, eight showed neuroforaminal stenosis and four had both central canal and neuroforaminal stenosis. Two patients (10.5 %) presented with motor deficits in the left limb due to neuroforaminal stenosis. Additional symptoms at initial presentation included back pain without leg pain in 26.3 %, leg pain attributed to neuroforaminal stenosis in 68.4 % of cases, and sensory deficits in 26.3 %, primarily manifesting as paresthesia. No cases of bowel or bladder dysfunction were observed. Nine of all patients (47.4 %) presented with spinal claudication resulting in a decreased pain-free walking distance (Fig. 1). Common coexisting conditions included high blood pressure in 47.4 % of cases, other cardiovascular diseases (such as atrial fibrillation and coronary artery disease) in 36.8 % and a history of cancer in 15.7 %. Among the 19 patients, eight (42.1 %) were on anticoagulant therapy which was paused seven days prior to the surgery.

### 3.2 Procedural characteristics

In 19 SA-XLIF procedures different spinal levels were addressed. The maximum number of levels treated was three performed in 31.6 % of cases. However, most patients (42 %) were treated at a single level. The remaining procedures were performed at two levels (26.3 %). The most commonly fused segment was L3/4 with 14 procedures (73.7 %), followed by L2/3 and L4/5 in nine procedures each (47.4 %). Intraoperative navigation with computed tomography (CT) was used in all procedures for the intraoperative scanning to confirm accurate positioning.

The choice of approach side (left or right) was influenced by anatomical features, such as access to the scoliotic spine's concave side. In the presence of central canal stenosis or spondylolisthesis the decision was based on the side of pain of radicular symptoms - often associated with foraminal stenosis - as well as anatomical characteristics and any previous surgeries in the relevant anatomical region. The cage was inserted through the left side in nine cases (47.4 %) cases and through the right side in 11 cases (52.6 %).

Bone morphogenetic proteins (BMPs) were used in 12 procedures (63.2 %). The remaining seven patients (36.8 %) had contraindications to BMPs, mostly due to a prevalent history of cancer. In altogether seven cases (36.8 %), patients underwent subsequent dorsal decompression; in one of these instances, the second decompression was not initially planned but was scheduled post-discharge due to the recurrence of symptoms after 195 days. In three cases (15.8 %) neurolysis or foraminal decompression was performed during the same surgery.

The median time until the second dorsal decompression within the same hospital stay was 5.5 days (IQR 3.75 days). Median surgery duration of the SA-XLIF-procedure was 106 min (IQR 52 min), while median blood loss was reported to be 200 mL (IQR 200 mL). Patients stayed in the hospital for a median time of eight days (IQR 10 days). Seventeen (89.5 %) of all patients were discharged home, while two (10.5 %) were transferred to a rehabilitation clinic.

### 3.3 Clinical outcome and complications

Of the 19 patients, 10 (52.6 %) experienced immediate relief of radicular symptoms after stand-alone XLIF with or without foraminal decompression or neurolysis. Additional dorsal direct decompression was required in 36.8 % of cases. Overall, 17 patients (89.5 %) achieved symptom relief after SA-XLIF, with or without additional dorsal decompression.

Two patients (10.5 %) required additional posterior instrumentation and deformity correction due to persistent symptoms. One patient underwent secondary instrumentation 21 days post-operatively during the same hospitalization; secondary dorsal decompression was not considered due to existing posterior instrumentation at adjacent levels. The second patient was discharged and subsequently readmitted 58 days after SA-XLIF for posterior fixation due to secondary cage subsidence associated with a suspected low-grade infection. Although no pathogen was identified, histopathology confirmed low-grade, non-florid chronic inflammation.

Additional complications were reported in three cases (15.7 %): Two patients developed a postoperative epidural hematoma after secondary dorsal decompression, resulting in increased pain and mild new neurological deficits. The hematoma was surgically evacuated in both cases, and the patients improved with no persisting symptoms or deficits. In another patient, intraoperative CT imaging revealed cage misplacement, prompting an immediate revision procedure to reposition the cage correctly. The same patient also required two additional revision surgeries following secondary dorsal decompression due to a suspected CSF leak, which was intraoperatively identified as a postoperative seroma.

In summary, unplanned surgical interventions were necessary for six out of the 19 patients (31.6 %). In addition, no cases of ASD were observed in our cohort. However, the limited retrospective observation period may not fully capture longer-term degenerative changes.

### 3.4 Radiographic outcome

**Fig. 2:**
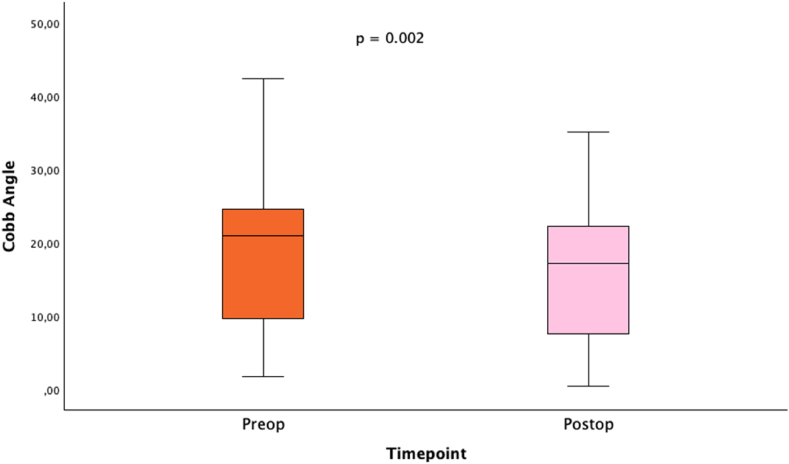
Comparison of preoperative and postoperative Cobb angles with significant reduction.

Foraminal height restoration was measured by comparing the preoperative and postoperative Cobb angles. A reduction in Cobb angle was achieved in all patients, with a decrease from a mean of 19° preoperatively to 15.9° postoperatively (mean delta Cobb: 3°, p = 0.002, paired *t*-test, Fig. 2). Sagittal vertical axis (SVA) measurements were available for 11 patients preoperatively and for only two patients postoperatively. Preoperative median SVA was 58 mm (range 26 mm–88 mm) with a positive sagittal imbalance.

### 3.5 Predictive factors for surgical success or failure

We performed statistical analysis to identify possible predictive factors for surgical success defined as the patient experiencing symptom relief without the need for further posterior instrumentation. We analyzed the relationship between initial diagnosis, number of levels fused, and surgical success rate. In the logistic regression model, initial diagnosis factors like ADS, listhesis, and stenosis types (central canal and neuroforaminal) showed low coefficients, indicating limited predictive value for surgical success in this cohort and no significant correlation (p = 0.998).

A moderate positive association (coefficient: 0.24) between preoperative Cobb angles, as well as postoperative Cobb angles (coefficient: 0.32) with success rate was detected. However, the change in Cobb angles was not significantly associated with a positive outcome (p = 0.44, student's t-test). Regarding the surgical procedure, the number of fused levels, SA-XLIF for two and three levels was associated with the highest success rate (100 %) as no patient underwent secondary surgery, while fusion at one level showed higher failure rates (23.4 %). However, the p-value in the logistic regression analysis was 0.998, indicating no significant association between the number of fused levels and success rate. Additional logistic regression analysis showed no significant association between age (p = 0.982) or sex (p = 0.999) and surgical success (Table 1).

**Table 1:** Odds ratios of surgical success from logistic regression model.

Factor	Odds ratio	P (chi-square test)
Adult degenerative scoliosis	1.00	1.0
Central canal stenosis	1.00	0.68
Neuroforaminal stenosis	1.00	1.0
No of fused levels	2.24	0.998
Foraminal decompression in 1st surgery	1.67	1.0
Cobb preoperative	1.12	0.24
Cobb postoperative	1.12	0.22
Delta Cobb preoperative-postoperative	1.25	0.44

*Note*: The SA-XLIF procedure was performed based on clinical indications. Consequently, the presence of ADS, central canal stenosis, or neuroforaminal stenosis is not expected to significantly influence surgical outcomes, as these conditions were prevalent in the majority of the cohort.

### 3.6 Case report: 71-year-old male patient presenting with right L4-radiculopathy

**Fig. 3:**
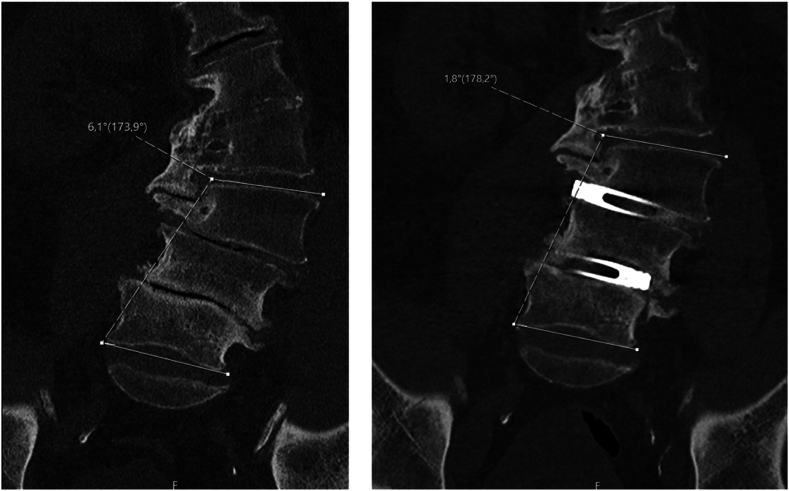
Preoperative (left) and postoperative (right) CT scans of the lumbar spine in coronal view including measurement of the Cobb angle.

A 71-year-old male patient presented with right-sided L4-ischialgia, primarily manifesting as activity-dependent pain without neurological deficits or disturbances in bladder and bowel function. His relevant medical history included sigma diverticulitis and urothelial carcinoma. Imaging revealed lumbar ADS, persistent spinal canal stenosis, and neuroforaminal stenosis at L3/4 and L4/5 on the right side, caused by scoliosis, despite prior surgeries: a right-sided hemilaminectomy at L5 in June 2017, right interlaminar fenestrations at L2/3 and L3/4 in February 2009, and a right-sided hemilaminectomy at L3 and L4 in 2008. Due to the persistent symptoms, surgery was scheduled for September 2021, including stand-alone XLIFs at L3/4 and L4/5 performed from the right side with intra-operative CT guidance and BMP application. Postoperatively, the patient showed significant improvement with resolution of his L4 radicular pain. He had no new neurological deficits and was discharged home on postoperative day 2. A reduction in the Cobb angle of 4.3° (from 6.1° to 1.8°) was observed on a postoperative CT scan (Fig. 3).

## 4 Discussion

We evaluated the effectiveness of stand-alone XLIF procedures in patients with neurogenic claudication and/or radicular symptoms due to adult spondylolisthesis or ADS. Of the 19 patients, only two (10.5 %) required secondary dorsal instrumentation due to persistent symptoms, primarily radicular pain. An additional 36.8 % underwent secondary dorsal decompression following the SA-XLIF procedure. This two-stage approach was preoperatively discussed with patients as part of the surgical planning, as indirect decompression may not always achieve sufficient neuroforaminal decompression in all cases, which emphasizes the importance of careful patient selection and preoperative counseling regarding the potential for staged procedures.

In our cohort, the median operative time of 106 min and the median intraoperative blood loss of 200 mL were comparatively low. Typically, the median blood loss for a three-level posterior lumbar instrumentation is reported to be between 500 and 1.200 mL^13^. Operating times for dorsal instrumentation techniques generally range from 180 to 300 min, depending on factors such as case complexity, surgeon experience, and whether the surgical approach is open or minimally invasive (Chen et al., 2024).

Regarding predictive factors, we identified a non-significant higher risk group for surgical failure: Patients who did not undergo neurolysis or decompression of the foramen during the first procedure (SA-XLIF). Nonetheless, none of the analyses yielded statistically significant results. Furthermore, our analysis shows that fusing more than one level may be associated with optimal outcomes, suggesting a balanced approach between minimal intervention and sufficient stabilization.

The analysis indicates that even more extensive lateral approaches, such as three-level SA-XLIF without screw augmentation is a viable approach, as it was not associated with lower success rates. We also identified a moderate positive association (odds ratio 1.12) between higher preoperative Cobb angles and success rate. This suggests that cases with a higher initial deformity are still likely to achieve a successful outcome, possibly due to the corrective potential of surgery. However, Cobb angle change alone was not significantly associated with the likelihood of surgical success (p = 0.44).

Finally, the question arises whether two less invasive procedures – stand-alone XLIF followed by dorsal decompression when needed – offer an advantage over a single, more extensive procedure involving posterior instrumentation and decompression. The revision rate of the posterior instrumentation method has been reported to be higher compared to the stand-alone XLIF, likely due to the complexity of the procedure and the potential for complications such as pseudarthrosis, misplacement of screws with neurological deficits, and hardware failure (Rodgers et al., 2011; Okuda et al., 2006). While revision rates for posterior instrumentation are reported to be around 10–20 % those for stand-alone XLIF are generally lower, ranging between 5 and 10 % (Kandwal et al., 2017). Given that both SA-XLIF and secondary dorsal decompression are relatively minor procedures, we believe this two-stage approach offers a clinical advantage, particularly for elderly patients. Although SA-XLIF alone provided symptomatic relief in the majority of patients (52.6 %). a significant proportion (36.8 %) required secondary dorsal decompression. This highlights the limitations of indirect decompression in selected cases and underscores the need for thorough preoperative assessment to identify candidates who may benefit from a planned two-stage strategy. In our cohort, many patients were initially considered for such an approach, with the second procedure reserved for those with persistent symptoms after SA-XLIF.

In conclusion, dorsal microsurgical decompression is a relatively short procedure typically taking 45–60 min. SA-XLIF is also shown to have a low risk profile as evidenced by a low complication rate of 10.5 %. Future research should investigate whether combining SA-XLIF and dorsal decompression in a single session with intraoperative repositioning could further improve outcomes and efficiency.

### 4.1 Limitations

This study has some limitations: First, it is a single-center study which may limit the generalizability of the results. A multicenter approach would have provided a larger representation and a more diverse patient population. Second, the sample size is relatively small with only 19 patients which reduces the statistical power. A larger cohort would increase the reliability of the results. In addition, the patient cohort was heterogeneous with some patients having undergone multiple prior lumbar surgeries which may have affected the results. Furthermore, we did not include a comparison group, such as a cohort that underwent dorsal posterior instrumentation which limits the ability to directly compare outcomes between the groups.

Most importantly, no structured follow-up was conducted beyond hospital discharge. Clinical improvement was assessed based on documented symptom relief at discharge and only re-hospitalizations at our institution were captured retrospectively. No systematic outpatient evaluations, patient-reported outcome measures, or further patient contacts were performed due to the retrospective observational design of the study. Therefore, some patients may have experienced persistent or recurrent symptoms that were either not reported or managed elsewhere. As a consequence, our findings primarily reflect the immediate postoperative status and perioperative complication profile, rather than providing robust long-term data. Future prospective studies with systematic long-term follow-up and standardized outcome assessments are required to better define the true clinical value and durability of stand-alone XLIF in this population.

Postoperative spinal fusion following SA-XLIF was not determined radiographically. In addition, the observed mean improvement in Cobb angle of 3° should be interpreted with caution, as this minimal change falls within the known range of variability in radiographic spinal assessments and may diminish over time.

However, imaging was not considered necessary in asymptomatic patients who reported no postoperative discomfort as clinical improvement is often consistent with successful fusion outcomes. Future studies should include routine imaging to better understand fusion rates and their impact on clinical outcomes as well as degenerative changes such as ASD.

### 4.2 Conclusions

In conclusion, SA-XLIF for ADS and spondylolisthesis appears to be a viable alternative to extensive posterior instrumentation in most patients. The median operative time was less than 2 h with minimal blood loss, making it particularly suitable for elderly and frail patients who may not tolerate longer, more invasive procedures. No statistically significant predictive factors were identified, suggesting that SA-XLIF without posterior instrumentation may be feasible for all patients, regardless of the number of fused levels or the degree of degeneration assessed by Cobb angles. However, as no structured long-term follow-up was performed, the current findings primarily reflect the immediate postoperative outcomes at hospital discharge. Prospective studies with systematic long-term assessments are necessary to further validate the effectiveness and durability of this approach.

## Availability of data and materials

The source data are stored at the Department of Neurosurgery, Technical University of Munich, Germany. To access the raw data or to discuss potential collaboration, please contact the corresponding author directly.

## Author contributions

CA: Conception and design, Data acquisition, Analysis, Drafting the article, Final approval.

MS: Conception and design, Revising the article for important intellectual content, Final approval.

PB: Conception and design, Revising the article for important intellectual content, Final approval.

VB: Conception and design, Analysis, Final approval.

BM: Conception and design, Revising the article for important intellectual content, Final approval.

## Declaration of generative AI and AI-assisted technologies in the writing process

The authors used ChatGPT-4o (OpenAI, San Francisco, California, USA) to assist with spelling, grammar, and style checks during the preparation of this work. Following this process, they thoroughly reviewed and revised the content as necessary and take full responsibility for the final published version.

## Funding

No funding for research, writing, and/or publication of this article was received by the author(s).

## Declaration of competing interest

The authors declare the following financial interests/personal relationships which may be considered as potential competing interests:Bernhard Meyer reports a relationship with Spineart Deutschland GmbH (Frankfurt, Germany) that includes: board membership and consulting or advisory. Bernhard Meyer reports a relationship with Medacta International (Castel San Pietro, Switzerland) that includes: consulting or advisory. Bernhard Meyer reports a relationship with Sonovum GmbH (Leipzig, Germany) that includes: board membership and equity or stocks. Bernhard Meyer reports a relationship with Medtronic (Meerbusch, Germany) that includes: board membership and consulting or advisory. Bernhard Meyer reports a relationship with Icotec AG (Altstätten, Switzerland) that includes: board membership and consulting or advisory. Bernhard Meyer reports a relationship with Zeiss (Oberkochen, Germany) that includes: board membership and consulting or advisory. Bernhard Meyer reports a relationship with DePuy Synthes (West Chester, PA, USA) that includes: consulting or advisory. Bernhard Meye reports a relationship with Brainlab AG (Munich, Germany) that includes: board membership and consulting or advisory. Vicki M. Butenschoen reports a relationship with Brainlab AG (Munich, Germany) that includes: consulting or advisory. Bernhard Meyer reports a relationship with Ulrich Medical (Ulm, Germany) that includes: board membership and consulting or advisory. If there are other authors, they declare that they have no known competing financial interests or personal relationships that could have appeared to influence the work reported in this paper.
